# Meta-analysis of remote ischemic conditioning in patients with acute myocardial infarction

**DOI:** 10.1038/srep43529

**Published:** 2017-03-08

**Authors:** Changfeng Man, Dandan Gong, Yongjing Zhou, Yu Fan

**Affiliations:** 1 Institute of Molecular Biology & Translational Medicine, the Affiliated People’s Hospital, Jiangsu University, Zhenjiang, Jiangsu, (212002) PR China

## Abstract

Effects of remote ischemic conditioning (RIC) in acute myocardial infarction (AMI) patients remain conflicting. We performed this meta-analysis of randomized clinical trials (RCTs) to evaluate the benefits of the RIC in patients with AMI. Potentially relevant RCTs were identified by searching PubMed, Embase, Cochrane Library, VIP, CNKI, and Wanfang database until November 2016. RCTs evaluating RIC using intermittent limb ischemia-reperfusion in AMI patients were included. Thirteen RCTs were identified and analyzed. Meta-analysis showed that RIC significantly reduced the area under the curve (AUC) of creatine kinase-myocardial band (CK-MB) (standardized mean difference [SMD] −0.29; 95% confidence intervals [CI] −0.44 to −0.14; *P* = 0.0002) and AUC of troponin T (SMD −0.22; 95% CI −0.37 to −0.08; *P* = 0.003). Risk ratio (RR) for ≥70% ST-segment resolution favored RIC group than the control group (RR 1.39; 95% CI 1.03–1.86; *P* = 0.03). RIC also significantly reduced all-cause mortality (RR 0.33; 95%CI 0.17–0.64; *P* = 0.001). Subgroup analyses on the CK-MB AUC and ST-segment resolution ≥70% rate showed that the effects of RIC appeared to be affected by the limb used, duration of RIC, and clinical setting. RIC may offer cardioprotective effects by improving ST-segment resolution and reducing the infarct size in AMI patients.

Percutaneous coronary intervention (PCI) and thrombolysis are well established reperfusion strategies in patients with acute myocardial infarction (AMI). Despite timely reperfusion approaches, the morbidity and mortality of AMI remain higher. Early reperfusion of occluded artery of myocardium is considered the most effective methods to minimize infarct sizes. However, abrupt restoration of blood flow may cause myocardial ischemia–reperfusion injury, leading to enlarge the infarct size^1^. Currently, there are no effective therapeutic interventions against myocardial reperfusion injury^2^.

Remote ischemic conditioning (RIC) induced by ischemia in a distant organ is a promising approach in the prevention of myocardial ischemia–reperfusion injury^3^. There are an increasing number of clinical trials evaluating cardioprotective effects of the RIC in AMI patients. A number of studies have demonstrated the cardioprotective effects of RIC in terms of improved myocardial perfusion and reduced infarct size in patients undergoing primary PCI^4^,^5^,^6^,^7^,^8^,^9^,^10^,^11^,^12^ or thrombolysis^13^,^14^,^15^,^16^ with conflicting findings. Furthermore, no previous meta-analysis has specifically focused on the cardioprotective effects of RIC in patients with AMI.

Hence, we aimed to evaluate the possible cardioprotective effects of RIC induced by intermittent limb ischemia–reperfusion in patients with AMI by conducting a meta-analysis of randomized clinical trials (RCTs).

## Results

### Literature search and study characteristics

**Figure 1:**
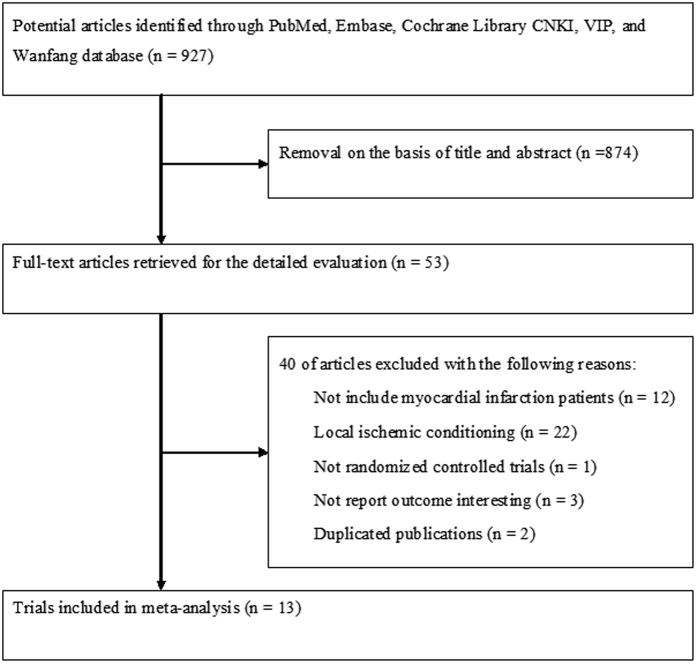
Flow chart of the literature search.

**Figure 2:**
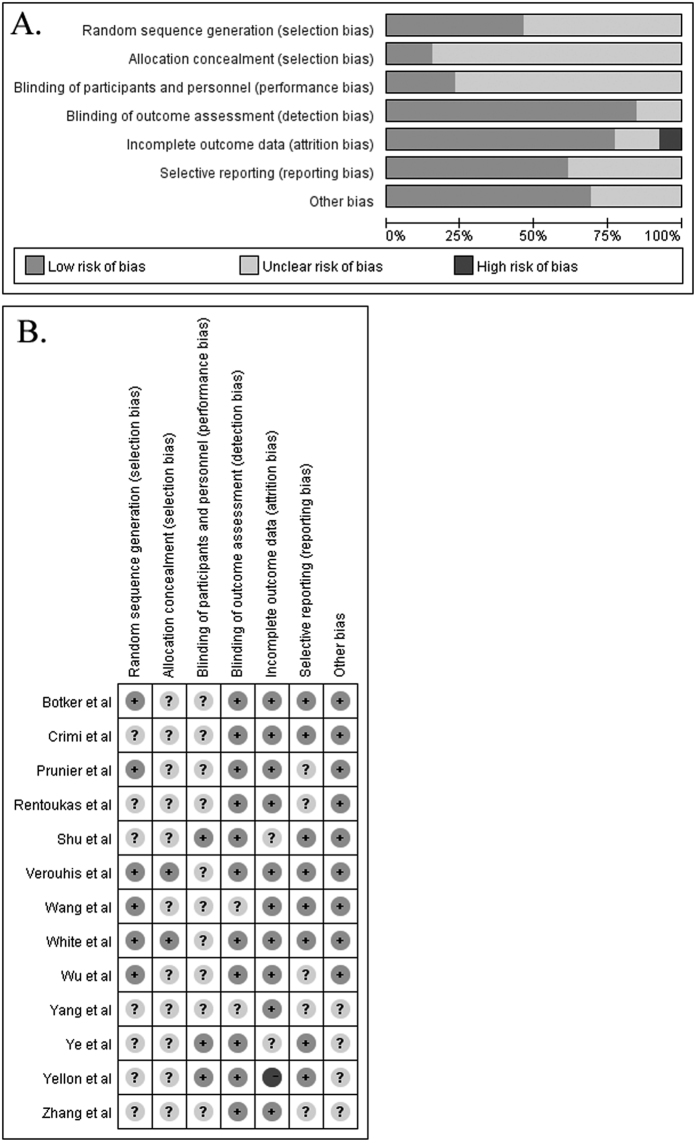
Risk of bias graph (**A**) and risk of bias summary (**B**).

The initial literature search produced 927 potential records. After reviewing the titles and abstracts, 874 records were removed. A total of 53 potentially eligible full-text articles were retrieved for the eligibility. After application of our predefined inclusion criteria, 13 articles^4^,^6^,^7^,^8^,^9^,^10^,^12^,^13^,^14^,^15^,^16^,^17^,^18^ were eventually included in the quantitative meta-analysis (Fig. 1). Tables 1 and 2 summarizes the characteristics and demographic data of the included trials. Of the 13 trials, 880 patients were randomized to RIC and 876 patients were allocated to the controls. Eight trials^4^,^6^,^7^,^8^,^9^,^10^,^12^,^18^ were performed in patients undergoing primary PCI, and 5 trials^13^,^14^,^15^,^16^,^17^ were conducted in patients receiving thrombolysis. All the eligible trials were published between 2006 and 2016. The sample size of the individual trials ranged from 35 to 519. RIC was performed by inflating a blood-pressure cuff placed on the arm in 9 trials, whereas 4 trials^8^,^9^,^17^,^18^ selected the leg. Two trials^6^,^7^ had less than a 30-minute duration of RIC and others had 30 minutes or over. Risk of bias of the included trials is shown in the Fig. 2.

### Infarct size as estimated by CK-MB and CK release

**Figure 3:**
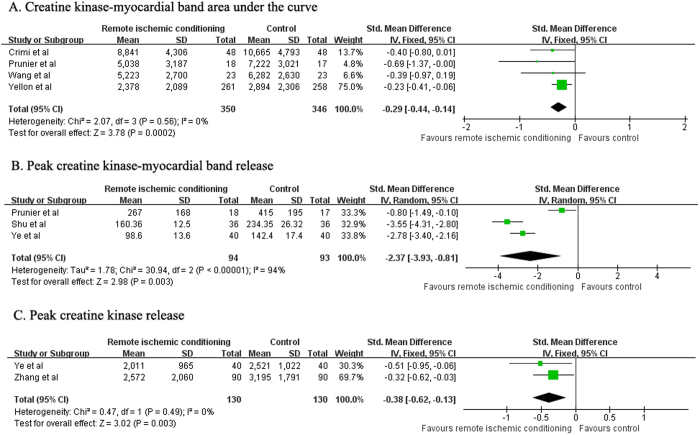
Forest plots for creatine kinase (CK)-MB area under the curve (**A**), peak CK-MB (**B**), and peak CK (**C**) with or without remote ischemic conditioning in patients with acute myocardial infarction.

Data about RIC on infarct size as estimated by CK-MB AUC were available in 4 trials^8^,^9^,^10^,^16^. As shown in Fig. 3A, RIC was associated with a significant reduction in the CK-MB AUC (SMD −0.29; 95% CI −0.44 to −0.14; *P* = 0.0002) in a fixed-effect model, with no evidence of heterogeneity (I^2^ = 0%; *P* = 0.56). Sensitivity analysis indicated that the omission of anyone trial at each time did not obviously change the pooled SMD and 95% CI. RIC significant reduced the peak CK-MB levels (SMD −2.37; 95% CI −3.93 to −0.81; *P* = 0.003) in 3 trials^10^,^15^,^17^ in a random effect model, with evidence of significant heterogeneity (I^2^ = 94%; *P* < 0.001). (Fig. 3B). Two trials^14^,^15^ reported data on peak CK release. As shown in Fig. 3C, RIC was also associated with a significant reduction in peak CK (SMD −0.38; 95% CI −0.62 to −0.13; *P* = 0.003) compared with control group in a fixed-effect model, with no evidence of heterogeneity (I^2^ = 0%; P = 0.49).

### Infarct size as estimated by troponin T and troponin I release

**Figure 4:**
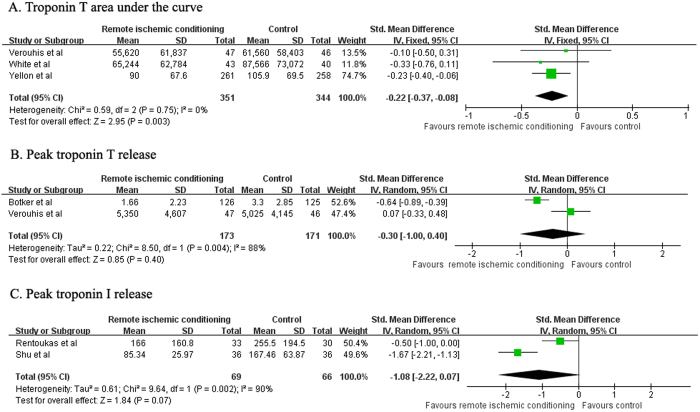
Forest plots for troponin T area under the curve (**A**), peak troponin T (**B**), and peak troponin I (**C**) with or without remote ischemic conditioning in patients with acute myocardial infarction.

Three trials^12^,^16^,^18^ reported troponin T AUC as outcome. As shown in Fig. 4A, RIC significantly reduced troponin T AUC (SMD −0.22; 95% CI −0.37 to −0.08; *P* = 0.003) compared with control group in a fixed-effect model, with no evidence of heterogeneity (I^2^ = 0%; *P* = 0.75). Peak troponin T data were reported in 2 trials^4^,^18^ and another 2 trials^6^,^17^ provided peak troponin I release data. However, there were no significant differences in peak troponin T (SMD −0.30; 95% CI −1.00 to 0.40; *P* = 0.40; Fig. 4B) and peak troponin I (SMD −1.08; 95% CI −2.22 to 0.07; *P* = 0.07; Fig. 4C) release between the RIC and the control group.

### Myocardial reperfusion injury as estimated by ST-segment resolution

**Figure 5:**
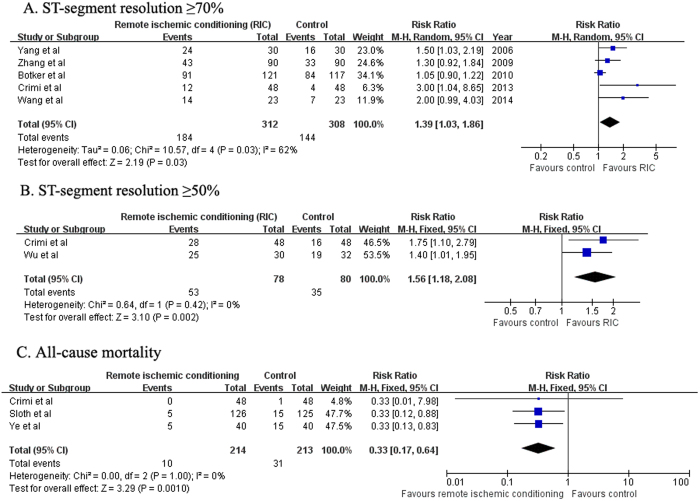
Forest plots for electrocardiographic ST-segment resolution ≥70% (**A**) and ST-segment resolution ≥50% (**B**), and all-cause mortality (**C**) with or without remote ischemic conditioning in patients with acute myocardial infarction.

Data about RIC on ST-segment resolution ≥70% were available in 5 trials^4^,^8^,^9^,^13^,^14^. As shown in Fig. 5A, the pooled RR for ≥70% ST-segment resolution favored RIC group (RR 1.39; 95% CI 1.03–1.86; *P* = 0.03) than the control group in a random effect model, with evidence of substantial heterogeneity (I^2^ = 62%; *P* = 0.03). In addition, the pooled RR was 1.25 (95% CI 1.09–1.44; P = 0.001) when we changed to a random-effect model. Effect of RIC on ST-segment resolution >50% was reported in two trials^7^,^8^. As shown in Fig. 5B, the pooled RR for ≥50% ST-segment resolution favored RIC group (RR 1.56; 95% CI 1.18–2.08; *P* = 0.002) than the control group in a fixed-effect model, with no evidence of heterogeneity (I^2^ = 0%; *P* = 0.42). The pooled RR was 1.51 (95% CI 1.15–1.97; *P* = 0.003) when we changed to a random effect model.

### All-cause mortality

Data about RIC on all-cause mortality were available in 3 trials^8^,^11^,^15^. As shown in Fig. 5C, RIC was associated with a significant reduction in all-cause mortality (RR 0.33; 95%CI 0.17–0.64; *P* = 0.001) in a fixed-effect model during the longest follow-up. There was no evidence of significant heterogeneity (I^2^ = 0%; *P* = 1.00).

### Subgroup analyses

Table 3 presents the detailed results of subgroup analysis. The effect of RIC on CK-MB AUC was stronger in patients undergoing PCI and RIC of the leg subgroups. RIC had a stronger effect on the rate of ST-segment resolution ≥70% in the leg (RR 2.36) than the arm (RR 1.16). Rate of ST-segment resolution ≥70% was significant in the patients treated with thrombolytic and RIC duration ≥30 min subgroups. However, the effects of RIC on ST-segment resolution ≥70% rate were not significant in patients undergoing PCI (RR 1.63; 95% CI 0.81–3.30; *P* = 0.17).

## Discussion

RIC is an easily feasible, well tolerated, and inexpensive technique^19^. A well-designed meta-analysis has evaluated the protective effects of RIC on myocardial injury and clinical outcomes^20^. However, there is high heterogeneity in the studied population, including ST-segment elevation myocardial infarction/urgent PCI, elective PCI, cardiac surgery, congenital heart disease repair, or coronary artery bypass graft. Moreover, this meta-analysis did not particularly address the cardioprotective effects of RIC on the AMI patients undergoing thrombolysis.

To the best of our knowledge, our meta-analysis specially focused on the cardioprotective effects of RIC induced by intermittent limb ischemia–reperfusion in AMI patients. Our meta-analysis of 13 RCTs involving patients with AMI treated by primary PCI or thrombolysis revealed that RIC induced by intermittent limb ischemia–reperfusion could limit the infarct size as estimated by CK-MB AUC, peak CK-MB release, and troponin T AUC. Moreover, RIC attenuated the myocardial reperfusion injury as estimated by improvement in ST-segment resolution rate.

Troponin was commonly used as a sensitive biomarker for early myocardial injury. In our pooled analysis, RIC significantly reduced troponin T AUC. However, no significant differences were observed between RIC and control group in terms of peak levels of troponin T or troponin I release. These findings may be explained by lack of statistical power due to small sample sizes included in the analysis.

Subgroup analysis showed that on the CK-MB AUC and ST-segment resolution ≥70% rate showed that the effects of RIC appeared to be affected by the limb used, duration of RIC, and clinical setting. RIC appeared to have a pronounced effect on the CK-MB AUC in patients undergoing primary PCI than thrombolysis (SMD −0.46 vs. −0.23). This finding may be explained by type of cardiac intervention may have different impacts on myocardium, and PCI itself may cause a higher release of cardiac biomarkers. ST-segment resolution has been recognized as a marker of efficient microvascular reperfusion. Resolution of ST-segment deviation after reperfusion is associated with better outcome after ST-segment elevation myocardial infarction^21^. By contrast, rate of ST-segment resolution ≥70% was significant in the patients treated with thrombolytic but not in patients undergoing PCI. However, interpretation of our findings should be cautioned due to the small number of trials in the stratified analysis.

This simple intervention is easily applied in AMI patients and may have the potential to reduce cardiac morbidity and mortality. Despite RIC could attenuate cardiac ischemic biomarker release, the effect of RIC on clinical endpoints is conflicting. Our pooled result showed that RIC was associated with a significant 67% reduction in all-cause mortality. However, this finding should be interpreted with caution because the patient numbers were relatively small as well as individual event numbers were low.

There is no standard protocol to induce RIC. Different protocols of RIC may have different cardioprotective effects^22^. RIC stimulus can be applied prior to the intervention, during ischemia, or after blood flow restoration. The timing and site could have potentially affected the cardioprotective effects of RIC. Loukogeorgakis *et al*. has demonstrated a dose-response protective effect with regard to number of cycles of RIC^23^. In order to achieve the maximal protective effect of RIC, sufficient threshold stimulus should be reached. Our subgroup analyses indicated that the effects of RIC on ST-segment resolution ≥70% rate were only statically significant in the RIC duration ≥30 min or by the lower limb subgroups. According to these findings, a RIC protocol of at least 3 cycles of 5 min ischemia and 5 min reperfusion (a total duration ≥30 min) particularly in the low limb is recommended.

Several potential limitations should be noted. First, this meta-analysis was not based on patient-level data. The potential impact of individual patient data including age, hypertension, diabetes, dyslipidaemia or medications cannot be excluded. Second, infarct size was determined at different time points with a certain degree of clinical heterogeneity. Third, subgroup analysis results were based on the limited number of trials and the small sample size, so these results should be further validated by more well-designed trials. Fourth, apart from all-cause mortality, we did not assess other clinical endpoints because they were only reported in a minority of trials; however, CK-MB or troponin^24^, and ST-segment resolution^25^,^26^ as surrogate indicators can strongly predict clinical prognosis^24^. Fifth, we did not conduct the Begg’s and Egger’s tests to evaluate publication bias because the included trials were less than the recommended arbitrary minimum number. Finally, this meta-analysis could not determine the optimal protocol of RIC in AMI patients.

In conclusion, RIC induced by intermittent limb ischemia–reperfusion appears to reduce the infarct sizes (determined by AUC CK-MB and troponin T), myocardial reperfusion injury (estimated by ST-segment resolution), and all-cause mortality in AMI patients. However, these conclusions may be not reliable due to insufficient number of trials and the small sample size. More well-designed trials are needed to confirm the cardioprotective effects of RIC in clinical practice.

## Methods

### Search strategy

The present meta-analysis was performed in accordance with the Preferred Reporting Items for Systematic Reviews and Meta-analysis’ (PRISMA) guidelines^27^. The PubMed, Embase, Cochrane Library, VIP, CNKI, and Wanfang database were searched for studies that evaluated the benefits of RIC using intermittent limb ischemia-reperfusion in patients with AMI. The following search terms were used: (RIC OR remote ischemic/ischaemic preconditioing OR remote ischemic/ischaemic perconditioning OR remote ischemic/ischaemic postconditioning AND myocardial infarction OR AND thrombolysis OR percutaneous coronary intervention OR coronary intervention AND randomized controlled trials OR RCTs. The latest update for literature research was done on November 28, 2016. Additional possible relevant trials were retrieved through a manual search of reference of the included articles.

### Study selection

Trials were considered eligible if they satisfied the following inclusion criteria: (1) RCTs comparing RIC versus no conditioning in patients with AMI; (2) patients were treated by primary PCI or thrombolysis; (3) RIC was induced by intermittent limb ischemia–reperfusion; and (4) trials at least reported one of the following outcome measures, including enzymatic myocardial infarction size as assessed by serum peak creatine kinase (CK), peak creatine kinase-myocardial band (CK-MB), CK-MB area under the curve (AUC) as well as troponin I, troponin T or troponin T AUC, electrocardiographic ST-segment resolution (≥50% or %70%), and all-cause mortality during the follow-up period. In addition, for the multiple publications from the same population, we chose the article with the complete data. Trials were excluded when: (1) trials consisted of no-AMI patients; (2) trials without reporting any of the outcomes interesting; and (3) non-randomized trials.

### Data extraction and quality assessment

Two investigators (CF Man and DD Gong) independently collected data from the included trials. Any disagreements between two reviewers were resolved by consensus. The extracted data included: the first author’s surname, year of publication, patients’ characteristics, RIC protocol, and outcome measures. For any missing or unclear data, we contacted the correspondence author by e-mail or telephone. The methodological quality of trials was assessed using Cochrane risk of bias tool of RCTs^28^, and grouped as low risk of bias, high risk of bias or unclear risk of bias.

### Data analysis and synthesis

All analyses were conducted using STATA statistical software version 12.0. The pooled effect sizes were calculated comparing the RIC to without conditioning, and summarized as a risk ratio (RR) with corresponding 95% confidence interval (CI) for dichotomous data and standardized mean difference (SMD) with 95% CI for continuous data. If continuous data were reported as median ± interquartile range (IQR), the mean and standard deviation (SD) were estimated using the median and the estimator SD = IQR/1.35^28^. Statistical heterogeneity across trials was evaluated using the Cochran’s Q test and I^2^ statistic. A *P*-value of Cochran’s Q test <0.10 or I^2^ statistic ≥50% represented significant heterogeneity. A random-effects model was selected when significant heterogeneity was observed; otherwise, a fixed effect model was used^29^. Subgroup analyses were performed by clinical setting (PCI vs. thrombolysis) and limb used (arm vs. leg). Sensitivity analysis was performed by sequentially deleting anyone study at each turn or replaced by the opposite statistical model to test the reliability of the pooled effect sizes.

## Additional Information

**How to cite this article**: Man, C. *et al*. Meta-analysis of remote ischemic conditioning in patients with acute myocardial infarction. *Sci. Rep.* **7**, 43529; doi: 10.1038/srep43529 (2017).

**Publisher's note:** Springer Nature remains neutral with regard to jurisdictional claims in published maps and institutional affiliations.

## Figures and Tables

**Table 1:** Demographic characteristic of the included studies.

Study/Year	Age (years) (RIC/Control)	%Male (RIC/Control)	Diabetes (RIC/Control)	Hypertension (RIC/Control)	Dyslipidaemia (RIC/Control)	Smokers (RIC/Control)
Yang *et al*. 2006^13^	63.9 ± 8.8	73.3%	NP	NP	NP	NP
Zhang *et al*. 2009^14^	63.2 ± 8.3 vs 63 ± 5.9	61% vs.61%	26% vs. 29%	31% vs.28%	47% vs.46%	49% vs.53%
Botker *et al*. 2010^4^	62.9 ± 12 vs. 63 ± 11	76% vs.75%	9% vs. 9%	38% vs.24%	15% vs.19%	56% vs.57%
Rentoukas *et al*. 2010^6^	62.9 ± 11.1 vs 61.2 ± 10.9	61% vs.60%	30% vs. 30%	48% vs.43%	48% vs.40%	73% vs.67%
Wu *et al*. 2011^7^	57.6 ± 7.6 vs 56.8 ± 8.9	70% vs.56%	20% vs. 1.6%	46.7% vs.53.1%	23.3% vs.40.6%	26.7% vs.18.8%
Ye *et al*. 2013^15^	45.7 ± 4.1	56%	NP	NP	NP	NP
Crimi *et al*. 2013^8^	61 ± 11 vs. 56 ± 11	85% vs.90%	9% vs. 15%	54% vs.53%	30% vs.33%	53% vs.54%
Wang *et al*. 2014^9^	63.1 ± 11.1 vs. 61.9 ± 14.7	73.9% vs.73.8%	30% vs. 26%	73.9% vs.56.5%	43.5% vs.30.4%	65.2% vs.52.2%
Prunier *et al*. 2014^10^	66.1 ± 16.2 vs. 61.7 ± 14.0	78% vs.76%	11% vs. 12%	50% vs.41%	33% vs.35%	22% vs.47%
Yellon *et al*. 2015^16^	57 ± 11 vs. 56 ± 11	80% vs.79%	43% vs. 40%	39% vs.43%	NP	21% vs.24%
White *et al*. 2015^12^	58 ± 10 vs. 61 ± 10	81.8% vs.77.6%	4% vs. 9%	22% vs.31%	27% vs.30%	47% vs.54%
Shu *et al*. 2016^17^	NP	NP	NP	NP	NP	NP
Verouhis *et al*. 2016^18^	61 (51–66) vs. 61 (57–68)	94% vs.96%	9% vs.9%	17% vs.28%	6% vs.7%	45% vs.30%

RIC, remote ischemic conditioning; NP, not provided.

**Table 2:** Baseline characteristics of the included studies.

Study/Year	Region	Clinical setting	Number of RIC/Control	Timing	RIC protocol			Outcome measures
					Limb	Cuff pressure	Cycles × I/R	
Yang *et al*. 2006^13^	China	AMI undergoing thrombolysis	30/30	During thrombolysis	Arm	NP	3 cycles × 5 min I and 5 min R	STR > 70%
Zhang *et al*. 2009^14^	China	AMI undergoing thrombolysis	90/90	During thrombolysis	Arm	NP	3 cycles × 5 min I and 5 min R	Peak CK, STR > 70%
Botker *et al*. 2010^4^	Denmark	STEMI undergoing primary PCI	126/125	Before/during PCI	Arm	200 mmHg	4 cycles × 5 min I and 5 min R	Peak troponin-T, STR > 70%, all-cause mortality^#^.
Rentoukas *et al*. 2010^6^	Greece	STEMI undergoing primary PCI	33/33	During PCI	Arm	>SBP 20 mmHg	3 cycles × 4 min I and 4 min R	Peak troponin-I
Wu *et al*. 2011^7^	China	STEMI undergoing primary PCI	30/32	Before PCI	Arm	250 mmHg	2 cycles × 5 min I and 5 min R	STR ≥ 50%
Ye *et al*. 2013^15^	China	AMI undergoing thrombolysis	40/40	During thrombolysis	Arm	NP	3 cycles × 5 min I and 5 min R	Peak CK, Peak CK-MB, all-cause mortality
Crimi *et al*. 2013^8^	Italy	Anterior STEMI undergoing primary PCI	48/48	During PCI	Leg	200 mmHg	3 cycles × 5 min I and 5 min R	72-h AUC CK-MB, STR > 50% or 70%, all-cause mortality
Wang *et al*. 2014^9^	China	STEMI undergoing primary PCI	23/23	Before PCI	Leg	200 mmHg	3 cycles × 5 min I and 5 min R	72-h AUC CK-MB, STR ≥70%,
Prunier *et al*. 2014^10^	France	STEMI undergoing primary PCI	18/17	During PCI	Arm	200 mmHg	3 cycles × 5 min I and 5 min R	2-h AUC CK-MB, peak CK-MB
Yellon *et al*. 2015^16^	UK	STEMI undergoing thrombolysis	261/258	Before/during thrombolysis	Arm	200 mmHg	4 cycles × 5 min I and 5 min R	24-h AUC CK-MB, 24-h AUC Troponin T
White *et al*. 2015^12^	UK	Anterior STEMI undergoing primary PCI	99/98	During PCI	Arm	200 mmHg	4 cycles × 5 min I and 5 min R	24-h AUC Troponin T
Shu *et al*. 2016^17^	China	STEMI undergoing thrombolysis	36/36	Before thrombolysis	Leg	>SBP 20 mmHg	3 cycles × 5 min I and 5 min R	Peak CK-MB, Peak troponin-I
Verouhis *et al*. 2016^18^	Sweden	Anterior STEMI undergoing primary PCI	47/46	Before/during PCI	Leg	200 mmHg	4 cycles × 5 min I and 5 min R	Peak troponin-T, 44-h AUC Troponin T.

RIC, remote ischemic conditioning; AMI, myocardial infarction; STEMI, ST-segment elevation myocardial infarction; I, ischemia; R, reperfusion; PCI, percutaneous coronary intervention; STR, ST-segment resolution; NP, not provided. ^#^Data from Sloth *et al*. 2014.

**Table 3:** Subgroup analyses on CK-MB AUC and ST-segment resolution ≥70%.

Subgroups	Number of trials	Pooled effect sizes	95% CI	Heterogeneity between trials	Treatment effect
CK-MB AUC					
Clinical setting					
PCI	3	SMD −0.45	−0.75 to −0.15	P = 0.750; I^2^ = 0.0%	P = 0.003
Thrombolysis	1	SMD −0.23	−0.41 to −0.06	—	P = 0.008
Limb used					
Arm	2	SMD −0.26	−0.73 to −0.07	P = 0.993; I^2^ = 0.0%	P = 0.002
Leg	2	SMD −0.40	−0.43 to −0.10	P = 0.193; I^2^ = 40.9%	P = 0.02
ST-segment resolution ≥70%					
Clinical setting					
PCI	3	RR 1.63	0.81 to 3.30	P = 0.02; I^2^ = 75.0%	P = 0.17
Thrombolysis	2	RR 1.39	1.08 to 1.79	P = 0.580; I^2^ = 0.0%	P = 0.01
Limb used					
Arm	3	RR 1.16	1.01 to 1.34	P = 0.140; I^2^ = 49.0%	P = 0.03
Leg	2	RR 2.36	1.30 to 4.29	P = 0.520; I^2^ = 0.0%	P = 0.005

Abbreviations: PCI, percutaneous coronary intervention; RR, risk ratio; WMD, weighted mean difference; CI, confidence interval; AUC; area under the curve; CK-MB, creatine kinase-myocardial band.
